# Short-Term Relationship between Hip Fracture and Weather Conditions in Two Spanish Health Areas with Different Climates

**DOI:** 10.1155/2015/395262

**Published:** 2015-02-10

**Authors:** José María Tenías, Marisa Estarlich, Eusebio Crespo, Carmen Román-Ortiz, Angel Arias-Arias, Ferran Ballester

**Affiliations:** ^1^Unidad Docente de Atencion Familiar y Comunitaria, Valencian School for Health Studies (EVES), 46017 Valencia, Spain; ^2^Spanish Consortium for Research on Epidemiology and Public Health (CIBERESP), Spain; ^3^University of Valencia, 46010 Valencia, Spain; ^4^Center for Research on Public Health (CSISP), 46020 Valencia, Spain; ^5^Service of Orthopedic Surgery and Traumatology, Hospital La Mancha Centro, 13600 Alcazar de San Juan, Spain; ^6^Research Support Unit, Hospital Mancha Centro, 13600 Alcazar de San Juan, Spain

## Abstract

*Objective*. To evaluate differences in the short-term relationship between weather conditions and the incidence of hip fracture in people aged 65 and over among two regions of Spain. *Methods*. Hip fracture incidence was calculated for the years 2000–2008 for residents of Health Area 14 in Valencian Community (Mediterranean climate) and the “Mancha Centro” Health Area in Castilla-La Mancha (inland climate), Spain. The relationship between hip fracture incidence and weather was analyzed with a case-crossover design and explored in subgroups defined by sex, age, and fracture type. *Results*. In the inland area, a positive and significant tendency for hip fracture incidence was observed (annual increase: 1.5%) whereas in the Mediterranean area a seasonal increase of 9% was noted in autumn and winter with respect to spring. Weather conditions, especially wind, were significantly associated with hip fracture incidence: days with more frequent windy periods and/or a greater wind velocity were associated with an increase in hip fracture incidence of 51% in the Mediterranean area and 44% in the inland area. *Conclusions*. Hip fracture incidence exhibits seasonal changes that differ between the Mediterranean and inland areas. The short-term relationship with climate, although similar in both areas, may partly explain these seasonal changes.

## 1. Introduction

Climate has been suspected of being a determining factor in the incidence of hip fractures for over 30 years, with changes in bone tissue quality compatible with osteomalacia being observed at certain times of year in cases of femoral neck-fractures [1]. Nevertheless, empirical studies exploring this association using original data on weather patterns and fracture incidence are all relatively recent and mostly conducted in northern latitudes, particularly in the Scandinavian countries [2–4], Canada [5, 6], the UK [7–10], and the USA [11–13]. Studies in this field conducted in either warmer climates [14–18] or the Southern Hemisphere [19, 20] are thus relatively scarce.

The role of seasonality in hip fracture incidence can be attributed either to the influence of weather and climate on bone metabolism or to the greater risk of falls associated with bad weather [21]. Evidence for the first hypothesis includes seasonal changes in levels of vitamin D and the parathyroid hormone (PTH) [22, 23], which may be related to the hours of sun exposure. Evidence for the second hypothesis includes the discovery of a short-term relationship (either on the same day or several days later) between the appearance of various meteorological phenomena and the occurrence of a hip fracture [2, 4, 5, 7–11, 15, 16, 20]. Only the second hypothesis, the greater risk of falls associated with bad weather, is considered in the present analysis.

The distribution of hip fracture incidence has been studied in the various autonomous communities of Spain [24], with the results presenting a great deal of variability between regions, both in the incidence of hip fractures and in the seasonal distribution of such incidents. In all regions, the incidence of hip fracture is greater in winter and lower in spring and summer. Nevertheless, seasonal variations are of a greater magnitude in regions with a warmer climate (the Canary Islands, Catalonia, the Valencian Community, Murcia, the Balearic Islands, and Andalusia) than in the colder regions in Northern and Central Spain. These differences may be due to a different relationship between weather conditions and hip fracture incidence, one that depends on the climate of each particular region. However the study by Alvarez-Nebreda et al. [24] did not evaluate possible reasons for the seasonal relationships and/or variability in hip fracture incidence across Spain, such as short-term relationships between climatic and weather variables and hip fracture incidence.

A previous study conducted in the Valencian Community [16] showed a positive trend (persistent, systematic tendency of a series) and marked seasonality for hip fracture incidence, with increases in the autumn and winter compared to spring and summer. An analysis of the short-term relationship between the duration and speed of the wind and hip fracture incidence through a case-crossover design indicated a positive and significant association. Thus, on days with a higher frequency of wind gusts compared to calmer days (comparing the top quartile of frequency of periods of calm wind to the bottom quartile), the risk of fracture increased by 32% (IC 95%: 10–58%), with this association being more marked in patients under 75 years of age than in those over 75. There are no published studies analyzing whether these associations occur in other regions of Spain, especially the inland regions, which have a continental climate with colder winters than the Mediterranean zone.

This study aims to explore the seasonality of hip fracture incidence and to ascertain whether the short-term relationship between weather conditions and hip fracture incidence is similar or different in two areas in Spain with distinct climates, namely, an area near the Mediterranean coast and an inland area with a continental climate.

## 2. Materials and Methods

**Figure 1:**
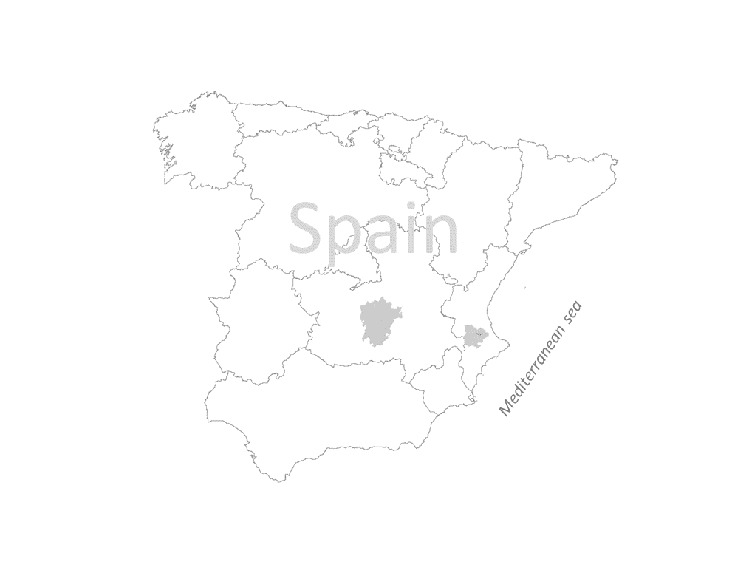
Location of the two Spanish areas analyzed. In the center, the “inland area” (reference hospital in Alcazar de San Juan, Ciudad Real, Autonomous Region of Castilla-La Mancha). Near the coast, the “Mediterranean area” (reference hospital in Xàtiva, Valencia, Valencian Community).

Our study was conducted in two health areas in regions in Spain with different climates (Figure 1). The first, which has a Mediterranean climate, corresponds to Health Area 14 of the Valencian Community, with a population of 185,002 inhabitants distributed throughout 64 municipalities in an area measuring 1874 km^2^. The other region is located inland on the southern Spanish subplateau and is known as the “La Mancha Centro” Health Area of Castilla-La Mancha. It has a population of 205,974 inhabitants spread throughout 35 municipalities over an area of 6006 km^2^ (Figure 1). The study period lasted 9 years, from January 1, 2000, to December 31, 2008.

For the main outcome or endpoint (incidence of hip fracture), we selected cases of osteoporotic hip fractures in patients 65 years of age or over who were admitted to a reference hospital in each of the regions during the study period. The main data source was the Basic Minimum Data Set (BMDS or Conjunto Mínimo Básico de Datos (*CMBD*) in its Spanish abbreviation) hospital registry. We chose area residents over 65 years of age who were admitted with a main diagnosis of hip fracture (ICD-9 820.0 to 820.9), excluding cases with a suspected diagnosis of a nonosteoporotic hip fracture (Paget's disease, chronic kidney failure, thyroid pathologies, neoplasms, connectivopathy, etc.) or as a result of a traffic accident. To calculate hip fracture incidence, we obtained demographic data (total population, gender, and age distribution) corresponding to the study period (2000–2008) and the respective health areas from the National Institute of Statistics (Instituto Nacional de Estadística (*INE*) in its Spanish abbreviation).

As independent or explanatory variables we collected the daily maximum, minimum, and median temperatures (in degrees Celsius); daily relative humidity (%); and daily precipitation (in milliliters per day). We also collected data on the number of days with weather phenomena such as the dew, frost, wind, hail, electrical storms, snow, and fog. Two wind variables, frequency of periods of calm (tenths of an hour) and the maximum wind speed (km per hour), were also recorded. These data, obtained from the State Meteorological Agency (*AEMET* in its Spanish abbreviation), came from the regional meteorological stations located in the cities of Xàtiva and Ontinyent, both in the Valencian Community, and from an additional two weather stations (“Alcazar de San Juan” and “Alcazar de San Juan-Las Perdigueras”) located in the municipality of Alcazar de San Juan in Castilla-La Mancha. For incomplete data series (less than 20%) we incorporated information from weather stations closest to the municipalities in question.

Time trend was defined as the number of years elapsed since the beginning of the time series whereas seasonality was represented as natural seasons (spring to winter).

### 2.1. Statistical Analysis

We described meteorological data as median (minimum–maximum of daily values) and weather phenomena days (snow, hail days with snow, storms, fog, dew, and frost) as percentages. We estimated hip fracture incidence as cases per 10,000 persons-year.

We explored the short relationship between hip fracture incidence and weather with a case-crossover design, taking the individual as the unit of analysis and establishing the index episode as the day of admittance to hospital. For each event (hip fracture), we assigned an exposure period (meteorological conditions on the same day or the day before) along with one or more control periods. We selected four control periods on the same day of the week, one and two weeks before and after the index episode (day of the hospital admittance). In this way, we attempted to control for weekly seasonality and the series tendency by using a symmetrical method [25]. Alternatively, a semimetric method was designed in which we randomly selected a control period one week before or after the event. This method has been shown to control for both tendency and seasonality [26]. We show associations as an odds ratio (OR) and its 95% confidence interval (IC 95%) estimated with conditional logistic regression models.

We carried out a time series analysis for fractures occurring on each day of the series. To this end a generalized linear model (GLM) of the beta negative binomial type was used with a logarithmic link into which variables representing the tendency and seasonality of the series were introduced. The calculated parameters indicate the increase or decrease in the number of cases per year elapsed from the start of the series and the relative changes in the incidence for the corresponding season with respect to that used as a reference (seasonality component).

In each analysis the possible modification of the effect (introduction of interaction terms according to subgroup) was studied with respect to the patient's gender, age (65–74 and >75 years of age), and fracture type (intracapsular and extracapsular). All calculations were carried out with the Stata program (StataCorp LP).

## 3. Results

During the study period, 3904 patients aged 65 and over were admitted to the reference hospitals for hip fractures (1987 in the Mediterranean area and 1917 in the inland area). Of these, 116 cases were discarded as suspected nonosteoporotic fractures (1 due to Paget's disease, 58 due to chronic kidney failure, 54 due to thyroid pathology, 6 due to automobile accidents, and 3 due to various causes). A total of 3788 patients were selected (1931 in the Mediterranean area and 1857 in the inland area), which means an overall incidence (in both areas taken together) of 654 cases per 10^4^ inhabitants per year (95% CI 625–683 cases/10^4^ inhabitants/year), with a lower incidence in men (350 cases per 10^4^ inhabitants/year; 95% CI 320 to 385 cases per 10^4^ inhabitants/year) than in women (887 cases per 10^4^ inhabitants/year; 95% CI 842 to 933 cases per 10^4^ inhabitants/year). The incidence was greater in the Mediterranean area than in the inland region, especially for women (Table 1). Patient profiles by age, gender, anatomic area of fracture, and length of hospital stay were very similar in both geographic regions.

### 3.1. Time Distribution of Incidence Rates

There was a positive time trend in both geographic regions, although this was greater and statistically significant in the inland area, which had an average annual increase in the number of cases of 1.5%. The role of seasonality was greater in the Mediterranean zone, which had an increased incidence of 9% in autumn and winter compared to spring, although this difference did not reach statistical significance (Table 2).

### 3.2. Description of Weather Data

The values and distribution of the various meteorological variables were those expected for the Mediterranean and inland climate types. The inland area presented a wider range of temperatures, lower median levels of relative humidity, and a greater number of days with fog, ice (frost and hail), or snow. In contrast, the Mediterranean area exhibited milder temperatures, with greater median levels of relative humidity and a higher number of days with dew (Table 3). The precipitation in both regions was characterized by the scarcity of rain, with somewhat more rain falling in spring and autumn. In both areas, autumn and winter were the seasons with the worst weather.

### 3.3. Case-Crossover Analysis

All calculations shown correspond to the bidirectional symmetrical method (the semimetric method showed similar but less precise results). The associations between weather phenomena and hip fracture events were similar in both areas with some important differences, especially wind (Table 4). Thus, in the Mediterranean area, on the windiest days (with gusts lasting longer than average), a marked increase in hip fracture incidence rate (OR 1.51; 95% CI 1.31–2.01) with respect to the calmest days was observed. In the inland area, an excessive hip fracture incidence rate was observed on days when the wind speed was higher than the mean (OR 1.44; 95% CI 0.88–2.46). In general, the increased risk of hip fracture incidence associated with days with more frequent and/or high speed winds was 23.3% (95% CI 7.7 to 41.1) in the Mediterranean area whereas it was slightly lower and not statistically significant (11.0%; 95% CI −14.0 to 43.9) in the inland region.

In the inland region, no increase was observed for snowy days (OR = 1.13; 95% CI 0.70–1.84) or on the rare days that the snow actually accumulated on the ground (OR = 1.25; 95% CI 0.61–2.53).

**Figure 2:**
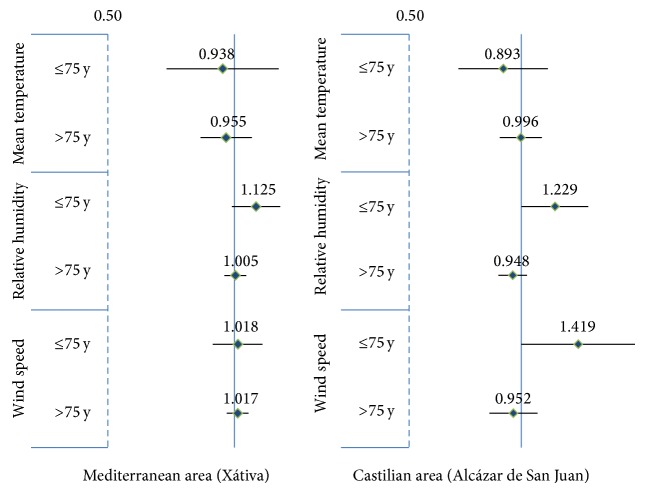
Association between temperature, humidity, and wind velocity and hip fracture incidence in two Spanish regions. Results represented as odds ratio per unit of change of one standard deviation of meteorological variable (7° Celsius, 10%, and 10 Km/h, resp.) and 95% confidence intervals in subgroups defined by patient age (cut-off: 75 years).

To facilitate comparison between the two areas we estimated the association between temperature, humidity, and wind velocity and hip fracture incidence as odds ratio per unit of change of one standard deviation of meteorological variable (7° Celsius, 10%, and 10 Km/h, resp.) in subgroups defined by patient age (cut-off: 75 years) (Figure 2).

The main differences observed in the subgroup analyses were those regarding subject age (over or under 75 years of age), with associations, although nonsignificant, that tended to be of greater magnitude in younger patients. This phenomenon was noted in both geographic regions. Subject gender and fracture type (intra- versus extracapsular) did not modify the associations observed.

## 4. Discussion

This study shows how adverse weather conditions may be associated with the incidence of hip fracture in two regions of the Iberian Peninsula. In general, the results were statistically similar in both regions, but in the Mediterranean area, in which a more notable seasonality for hip fracture incidence was observed, the findings were clearer with regard to the relationship of hip fracture incidence with weather phenomena, especially wind. Patient age plays a differential role in the relationship between hip fracture incidence and weather patterns in that younger patients show a stronger association with weather changes. This may be related to greater exposure since younger people spend more time outdoors than their older counterparts.

Our findings are thus consistent with the hypothesis that areas showing a higher level of seasonality also have the most marked relationship between hip fracture incidence and weather conditions.

In general, studies published to date that have been conducted either in our area or in other countries exhibit certain heterogeneity in their results. The noteworthy differences with respect to the methodology and analytical strategies used in these studies may explain, at least in part, many of the differences in results. Thus, while several studies use the day as the unit of analysis, others use greater time spans, usually one month [21]. Analytical methodologies also vary, from correlations and/or linear regression models that do not control for tendency or seasonality in time series to Poisson regression models to specific time series analysis methods such as ARIMA models [21]. Despite this heterogeneity, the results show a predominance of negative and significant associations between hip fracture incidence and temperature [5, 12, 17–20] and positive relationships with certain atmospheric phenomena such as snow [4–6, 11, 27] and wind [6, 11, 12, 19].

We have analyzed all three of these variables in our study. Although we found no statistically significant association for temperature, it did exhibit a negative association with hip fracture incidence of a magnitude similar to that observed in other studies. Moreover, variations in temperature may partly explain the seasonality of hip fracture incidence: a 7.7-degree increase in temperature (average temperature difference between spring and winter) would thus correspond to an increased individual risk of 8.0%, a percentage very similar to that calculated through comparisons of the difference in hip fracture incidence in these two seasons (9.3%).

As expected, snow was an uncommon event in our series, especially in the Mediterranean area, which led to inconsistent associations.

Wind has been analyzed in relatively few studies conducted in countries with a climate different from that of Spain, such as the USA [11, 12], Australia [19], and Canada [6]. Lau et al. found a greater monthly incidence of hip fracture in people over 75 years of age in months with a greater number of windy days, although this association disappeared when the seasonality of the series was controlled for or with the introduction of other weather variables [19]. Two studies carried out in the USA found a positive relationship between wind speed and monthly hip fracture incidence [12] and an increased risk (35% in individuals over 75 years of age) on days with high winds [11]. Likewise, in our study, the increased risk of hip fracture associated with days with more frequent winds and/or high speed winds was higher in the Mediterranean area whereas it was slightly lower and not statistically significant in the inland region.

The study conducted by Modarres et al. in Canada [6] analyzed the association between the monthly incidence of hip fracture and various meteorological variables and found no significant relationship between hip fracture incidence and wind speed. Even so, the relationship was positive and of a greater magnitude in the younger group of patients (40 to 74 years of age) than in patients aged 75 and over for both men and women.

Our study shows how within a single country, when different climate types are identified, the pattern of association between weather conditions and hip fracture incidence can vary. One of the advantages is that we have used the same design and data source for both the outcome (hospital admittance) and the exposure (weather variables) in the same time period and with the same analytical method. This means that the differences observed are most likely due to real differences in the association between climate and the health problem under study, namely, hip fracture. Seasonality may be further evidence of the existence of this relationship, since it can be explained to a great extent by the association between climate and fractures.

Still, our study has several limitations. For example, the temporal assignment of the event is problematic in that hospital admittance does not always coincide with the day the fracture occurred. However, in a pilot case series study conducted in the Mediterranean region, we were able to establish that 92.5% of emergency room visits were admitted to hospital on the same day the fracture occurred [16]. Another limitation entails the impossibility of distinguishing between and separately analyzing fractures caused by a fall and those due to other causes, although we assume that the vast majority (>90%) are caused by falls [28]. Finally, the difficulty in establishing whether the fall occurred within the home or outdoors should be noted as a limitation as it could affect the association with weather conditions, which would presumably be stronger for falls occurring outside the home. Studies conducted in similar environments have determined that the majority of falls (70%) occur within the home [29], a fact which may create a nondifferential bias that could lead to underestimation of the true effect of windy conditions.

In order to improve the design and development of preventative measures, future research should apply this methodology to other climate zones and incorporate data on where the event occurred (especially whether it occurred within the home or not) and the presence of other circumstances that could lead to a fall [13].

## Figures and Tables

**Table 1:** Description of hip fracture cases identified in two Spanish regions (2000–2008).

	Mediterranean area (*n* = 1931)	Inland area (*n* = 1857)	*P*
Incidence^a^ *n* (annual range)			
Global	654 (625 to 683)	559 (534 to 585)	<0.001
Males	350 (320 to 385)	310 (282 to 340)	0.07
Females	887 (842 to 933)	752 (713 to 792)	<0.001
Sex; *n* (%)			0.55
Female	1479 (76.6%)	1407 (75.8%)	
Male	452 (23.4%)	450 (24.2%)	
Average age (SD; range)	82.0 (7.1; 65 to 101)	81.9 (6.9; 65 to 103)	0.70
Categorized			0.36
65–74 y	297 (15.4%)	266 (14.3%)	
≥75 y	1634 (84.6%)	1591 (85.7%)	
Fracture site; *n* (%)			0.81
Intracapsular	587 (38%)	709 (38.4%)	
Extracapsular	957 (62%)	1136 (61.6%)	
Not specified	387	12	
Average hospital stay, days (SD; range)	10.8 (5.0; 1 to 83)	10.9 (9.0; 1 to 190)	0.60

^a^Nonstandardized rate in cases per 10^4^ persons-year.

**Table 2:** Time trend and seasonality of hip fracture incidence in two Spanish regions (2000–2008).

	Mediterranean area	Inland area
Tendency (per year)	1.004 (0.990–1.018)	1.015 (1.001–1.030)
Seasons		
Spring (ref.)	1	1
Summer	0.963 (0.866–1.07)	1.032 (0.934–1.140)
Autumn	1.094 (0.991–1.208)	1.050 (0.951–1.160)
Winter	1.093 (0.989–1.207)	0.884 (0.792–0.986)

Results expressed as incidence rate ratio (95% confidence interval); ref.: reference category.

**Table 3:** Meteorological descriptions of two Spanish areas (2000–2008).

Meteorological variables	Mediterranean area		Inland area	
	Annual values	Seasonal variations Spring/summer/fall/winter	Annual values	Seasonal variations Spring/summer/fall/winter
Mean temperature (°C)	17.9 (0.8 to 33.0)	18.7/26.4/15.7/11.0	14.4 (−4.5 to 31.7)	15.9/25.7/11.4/07.2
Mean relative humidity (%)	65.0 (32.5 to 98.4)	63.5/64.5/67.9/66.2	61.5 (9.1 to 99.0)	62.3/43.5/69.0/74.3
Precipitation (mL)	17.9 (0 to 3050)^*^	23.4/4.9/29.5/14.9	10.1 (0 to 507)^*^	15.6/2.5/13.4/8.7
Wind				
Periods of calm (tenths of an hour)	50 (1 to 239)	43/53/57/52	nd	nd
Maximum speed (km/h)	24 (7 to 86)	26/25/21/23	27 (3 to 70)	31/29/22/29
Other weather phenomena, *n* (%)				
Days with snow	3 (0.1%)	0/0/0.2%/0.4%	41 (1.2%)	0.2%/0/1.1%/3.7%
Days with hail	2 (0.1%)	0.4%/0/0/0	57 (1.7%)	3.6%/1.1%/0.9%/1.4%
Days with storms	59 (2.7%)	2.2%/6.2%/2.2%/0.2%	179 (5.4%)	11.2%/6.9%/2.7%/0.9%
Days with fog	29 (0.9%)	0/0.7%/1.5%/3.1%	335 (10.2%)	1.4%/0.4%/17.0%/22.3%
Days with dew	979 (44.7%)	28.4%/48.6%/55.3%/46.5%	430 (13.1%)	2.2%/7.0%/22.8%/10.3%
Days with frost	49 (2.2%)	0/0/2.6%/6.5%	378 (11.5%)	1.0%/0/14.7%/30.8%

Results expressed as median (minimum–maximum of daily values); ^*^mean (minimum–maximum); nd: not determined.

**Table 4:** Association between meteorological variables and occurrence of hip fracture: results of the case-crossover analysis.

	Lag	Mediterranean area	Inland area
Average temperature	0	0.993 (0.975–1.011)	0.997 (0.980–1.013)
	1	0.990 (0.971–1.008)	0.999 (0.982–1.015)

Average relative humidity	0	1.002 (0.997–1.008)	0.999 (0.991–1.007)
	1	0.997 (0.992–1.003)	0.994 (0.986–1.003)

Rain (yes versus no)	0	1.000 (0.999–1.001)	1.000 (0.999–1.002)
	1	0.874 (0.736–1.036)	0.915 (0.812–1.030)

Dew (yes versus no)	0	0.996 (0.869–1.141)	1.008 (0.859–1.184)
	1	1.019 (0.889–1.167)	1.083 (0.924–1.269)

Frost (yes versus no)	0	0.853 (0.550–1.321)	0.965 (0.780–1.165)
	1	0.997 (0.667–1.490)	1.009 (0.817–1.248)

Fog (yes versus no)	0	0.885 (0.509–1.537)	0.980 (0.812–1.183)
	1	1.080 (0.662–1.762)	0.966 (0.79–1.171)

Storm (yes versus no)	0	1.215 (0.855–1.727)	1.084 (0.872–1.348)
	1	1.376 (0.957–1.979)	1.054 (0.843–1.317)

Frequency of less windy periods	0	1.000 (0.999–1.001)	nd
	1	0.997 (0.995–0.999)	nd

Wind velocity	0	1.002 (0.996–1.007)	1.001 (0.988–1.015)
	1	1.006 (0.999–1.013)	1.001 (0.987–1.015)

Results expressed as odds ratio (confidence interval 95%). Lag: association with meteorological conditions on the same day (0) or the day before (1) hospital admission.

Average temperature (OR by increase of 1°C); relative humidity (OR by increase of 1%); less windy periods (OR by increase in a tenth of an hour of periods of calm); wind velocity (OR by increase in 1 km/h); nd: not determined.

## References

[1] Aaron J. E., Gallagher J. C., Nordin B. E. (1974). Seasonal variation of histological osteomalacia in femoral-neck fractures.. *The Lancet*.

[2] Lauritzen J. B., Schwarz P., McNair P., Lund B., Transbol I. (1993). Radial and humeral fractures as predictors of subsequent hip, radial or humeral fractures in women, and their seasonal variation. *Osteoporosis International*.

[3] Lofthus C. M., Osnes E. K., Falch J. A. (2001). Epidemiology of hip fractures in Oslo, Norway. *Bone*.

[4] Frihagen F., Stoen R., Hygen H. O., Nordsletten L., Lofthus C. (2011). Ice and snow in Oslo gave a marked increase in distal radius fractures, but not in hip fractures. *Osteoporosis International*.

[5] Levy A. R., Bensimon D. R., Mayo N. E., Leighton H. G. (1998). Inclement weather and the risk of hip fracture. *Epidemiology*.

[6] Modarres R., Ouarda T. B. M. J., Vanasse A., Orzanco M. G., Gosselin P. (2012). Modeling seasonal variation of hip fracture in Montreal, Canada. *Bone*.

[7] Parker M. J., Martin S. (1994). Falls, hip fractures and the weather. *European Journal of Epidemiology*.

[8] Chesser T. J. S., Howlett I., Ward A. J., Pounsford J. C. (2002). The influence of outside temperature and season on the incidence of hip fractures in patients over the age of 65. *Age and Ageing*.

[9] Atherton W. G., Harper W. M., Abrams K. R. (2005). A year's trauma admissions and the effect of the weather. *Injury*.

[10] Murray I. R., Howie C. R., Biant L. C. (2011). Severe weather warnings predict fracture epidemics. *Injury*.

[11] Jacobsen S. J., Sargent D. J., Atkinson E. J., O'Fallon W. M., Melton L. J. (1995). Population-based study of the contribution of weather to hip fracture seasonality. *The American Journal of Epidemiology*.

[12] Mirchandani S., Aharonoff G. B., Hiebert R., Capla E. L., Zuckerman J. D., Koval K. J. (2005). The effects of weather and seasonality on hip fracture incidence in older adults. *Orthopedics*.

[13] Bischoff-Ferrari H. A. (2011). The role of falls in fracture prediction. *Current Osteoporosis Reports*.

[14] Caniggia M., Morreale P. (1989). Epidemiology of hip fractures in Siena, Italy, 1975–1985. *Clinical Orthopaedics and Related Research*.

[15] Montero Furelos L. A., Colino Sánchez A. L., Trobajo de las Matas J. E., Quevedo García L. A. (2001). Hip fractures, seasonal variations and influence of climatological parameters. *Revista de Ortopedia y Traumatologia*.

[16] Tenías J. M., Estarlich M., Fuentes-Leonarte V., Iñiguez C., Ballester F. (2009). Short-term relationship between meteorological variables and hip fractures: an analysis carried out in a health area of the Autonomous Region of Valencia, Spain (1996–2005). *Bone*.

[17] Lin H.-C., Xiraxagar S. (2006). Seasonality of hip fractures and estimates of season-attributable effects: a multivariate ARIMA analysis of population-based data. *Osteoporosis International*.

[18] Chiu K. Y., Ng T. P., Chow S. P. (1996). Seasonal variation of fractures of the hip in elderly persons. *Injury*.

[19] Lau E. M. C., Gillespie B. G., Valenti L., O'Connell D. (1995). The seasonality of hip fracture and its relationship with weather conditions in New South Wales. *Australian Journal of Public Health*.

[20] Turner R. M., Hayen A., Dunsmuir W. T. M., Finch C. F. (2011). Air temperature and the incidence of fall-related hip fracture hospitalisations in older people. *Osteoporosis International*.

[21] Román O. C., Tenías J. M., Estarlich M., Ballester F. (2014). Systematic review of the association between climate and hip fractures. *International Journal of Biometeorology*.

[22] Pasco J. A., Henry M. J., Kotowicz M. A. (2004). Seasonal periodicity of serum vitamin D and parathyroid hormone, bone resorption, and fractures: the Geelong Osteoporosis Study. *Journal of Bone and Mineral Research*.

[23] Bischoff-Ferrari H. A., Can U., Staehelin H. B. (2008). Severe vitamin D deficiency in Swiss hip fracture patients. *Bone*.

[24] Alvarez-Nebreda M. L., Jiménez A. B., Rodríguez P., Serra J. A. (2008). Epidemiology of hip fracture in the elderly in Spain. *Bone*.

[25] Bateson T. F., Schwartz J. (1999). Control for seasonal variation and time trend in case-crossover studies of acute effects of environmental exposures. *Epidemiology*.

[26] Navidi W., Weinhandl E. (2002). Risk set sampling for case-crossover designs. *Epidemiology*.

[27] Bischoff-Ferrari H. A., Orav J. E., Barrett J. A., Baron J. A. (2007). Effect of seasonality and weather on fracture risk in individuals 65 years and older. *Osteoporosis International*.

[28] Olmos J. M., Martínez J., García J., Matorras P., Moreno J. J., González-Macías J. (1992). Incidence of hip fractures in Cantabria. *Medicina Clinica*.

[29] Formiga F., Lopez-Soto A., Duaso E. (2007). Differences in the characteristics of elderly patients suffering from hip fracture due to falls according to place of residence. *Journal of the American Medical Directors Association*.

